# Combination of a T cell activating therapy and anti-phosphatidylserine enhances anti-tumour immune responses in a HPV16 E7-expressing C3 tumour model

**DOI:** 10.1038/s41598-021-82108-4

**Published:** 2021-02-24

**Authors:** Brennan S. Dirk, Genevieve Weir, Tara Quinton, Olga Hrytsenko, Marianne M. Stanford

**Affiliations:** 1 IMV Inc, Dartmouth, NS Canada; 2 Department of Microbiology and Immunology, Dalhousie University, Halifax, NS Canada

**Keywords:** Cancer immunotherapy, Tumour immunology

## Abstract

DPX is a novel delivery platform that generates targeted CD8 ^+^ T cells and drives antigen-specific cytotoxic T cells into tumours. Cancer cells upregulate phosphatidylserine (PS) on the cell surface as a mechanism to induce an immunosuppressive microenvironment. Development of anti-PS targeting antibodies have highlighted the ability of a PS-blockade to enhance tumour control by T cells by releasing immunosuppression. Here, C57BL/6 mice were implanted with HPV16 E7 target-expressing C3 tumours and subjected to low dose intermittent cyclophosphamide (CPA) in combination with DPX-R9F treatment targeting an E7 antigen with and without anti-PS and/or anti-PD-1 targeting antibodies. Immune responses were assessed via IFN-γ ELISPOT assay and the tumour microenvironment was further analyzed using RT-qPCR. We show that the combination of DPX-R9F and PS-targeting antibodies with and without anti-PD-1 demonstrated increased efficacy compared to untreated controls. All treatments containing DPX-R9F led to T cell activation as assessed by IFN-γ ELISPOT. Furthermore, DPX-R9F/anti-PS treatment significantly elevated cytotoxic T cells, macrophages and dendritic cells based on RT-qPCR analysis. Overall, our data indicates that anti-tumour responses are driven through a variety of immune cells within this model and highlights the need to investigate combination therapies which increase tumour immune infiltration, such as anti-phosphotidylserine.

## Introduction

The ability to engage and activate T cells to tumour-associated targets has the potential to elicit strong anti-tumour responses in vivo^1–3^. DPX, previously known as Depovax, is a novel formulation which enhances immune responses in humans and animals^4,5^. DPX encapsulates cancer specific peptides into a no release depot composed of an oil diluent, which provides long-lasting reservoir of antigens in vivo^4,5^. DPX formulation containing the HPV16 E7 peptide target (DPX-R9F) has demonstrated efficacy in mice bearing HPV16 E7-expressing C3 tumours, and efficacy is enhanced following PD-1 blockade, which has been shown to reinvigorate T cells, and decrease exhaustion to enhance anti-tumour responses^6,7^. Preclinical studies have demonstrated DPX formulations induce robust cytotoxic T cell (CTL) responses, and generation of tumour infiltrating CTLs^8^. In recent human clinical trials, DPX formulated with five survivin-specific peptides resulted in robust immune responses directed at the tumour target, survivin^9^. Pre-clinical and clinical data demonstrates enhanced anti-tumour responses with checkpoint inhibitors and additional adjuvants thereby expanding the clinical use of DPX-based therapies^5,6,10^. The combination of DPX-based formulation with checkpoint molecules and immune modulators represent different ways to target immune cells and augment their activation status^7^. This formulation has been demonstrated to work in combination with low dose intermittent cyclophosphamide (CPA), a treatment designated to modulate immune responses^11^. The sequencing of DPX-based formulation and CPA has been investigated pre-clinically, and implemented in clinical trials for its benefits as an immune modulator^5,6^. However, this treatment has yet to be investigated in combination with a specific cancer-targeted therapy as a prospective way to enhance efficacy in tumour challenge models.

Phosphatidylserine (PS) is a molecule present on the inner leaflet of the plasma membrane in normal heathy cells^12^. Upon apoptosis, PS is ‘flipped’ to the outer leaflet of the plasma membrane, rendering it exposed to the extracellular environment^12^. Through a process termed efferocytosis, phagocytic cells uptake PS-expressing apoptotic membranes through a direct interaction with TIM receptors and an indirect interaction with TAM receptors^13^. To prevent an over-stimulated immune response to apoptotic cells, the uptake of apoptotic bodies is naturally anti-inflammatory. This process is driven through the ability of TIM and TAM receptors present on the surface of macrophages and dendritic cells to stimulate downstream expression of immune-suppressive cytokines such as transforming growth factor β (TGF-β) and interleukin-10 (IL-10)^14–16^. Within an otherwise healthy individual, this process maintains cellular homeostasis, and prevents diseases such as atherosclerosis and a variety of inflammatory diseases. However, in addition to being expressed on apoptotic cells, PS can also be abundantly expressed on malignant cells, and represents a prominent mechanism of immune evasion, which can negatively impact patient prognosis^17^.

Within the tumour microenvironment (TME), PS becomes expressed on the outer leaflet of the plasma membrane due to rapid cell division, dysregulated membrane trafficking pathways, and upregulation of cell death associated pathways^18^. Although PS expression constitutes a cellular ‘eat-me’ signal, cancerous cells often usurp the immunosuppressive capabilities of PS to enhance survival of the cells through multiple mechanisms. First, circulating PS-expressing extracellular vesicles can dampen T cell activation^15^. Second, phagocytic cells ingesting PS-membranes fail to become mature and functional antigen-presenting cells^15^. Lastly, anti-inflammatory signals such as TGF-β and IL-10 further prevent effective anti-tumour responses by other cytolytic cell types^15^.

Pre-clinical studies have recently demonstrated the efficacy of targeting PS as a potential avenue to enhance current cancer immunotherapies. In vivo studies using anti-PS antibodies (3G4, 2aG4 and mch1N11) demonstrated efficient targeting to malignant cells and tumour vasculature, resulting in enhanced anti-tumour responses^19,20^. Moreover, when provided in combination with radiotherapy, these antibodies improved the efficacy of chemo and radiation therapy by increasing antibody-dependent cytotoxicity of PS-expressing cells. Furthermore, combination of anti-PS antibodies with anti-CTLA-4 and anti-PD-1 resulted in the increased infiltration of cytolytic T cells and reduced immune suppressive cell types such as regulatory T cells, and myeloid derived suppressor cells^21^. Taken together, the combination of anti-PS antibodies with current and new immunotherapies is an attractive approach to combating malignant cells.

Using a tumour-bearing mouse model of C3 cells expressing the HPV16 E7 antigen, we demonstrate DPX-R9F/CPA in combination with PS-targeting antibodies enhances T cells, macrophages, and dendritic cell infiltration into the tumour microenvironment providing a rationale for combining these treatments.

## Methods

### Mice and tumour implantations

Pathogen free, 6–8 week old female C57BL/6 mice were purchased from Charles River Labs (St. Constant, QC, Canada). Mice were housed under top-filtered conditions and provided food and water ad libitum.

The C3 cell line was provided by Dr. Martin Kast, and was derived from C57BL/6 embryo cells transfected with HPV16 DNA and demonstrated to express an immunodominant peptide from HPV16 E7 in the context of MHC class I on the cell surface^22^. The C3 cell line was maintained in IMDM supplemented with 10% fetal bovine serum, 2% penicillin–streptomycin, 50 mM mercaptoethanol and 2 mM L-Glutamine. In all tumour studies mice were implanted with 3 × 10^5^ C3 tumour cells in the left flank on study day 0.

Tumour growth was measured with digital calipers twice weekly, and size was calculated by the formula (width^2^ x length)/2. In survival studies, mice were terminated when tumour volume reached ≤ 2000mm^3^, or when mice displayed signs of poor health such as dehydration, decreased activity, hunched posture, tumour ulcerations, or site of injection reactions. When endpoints were identified, mice were humanely euthanized per CCAC guidelines.

### Peptides

All peptides were synthesized by NeoMPS (San Diego, USA) with 90% purity. All studies used the peptide epitope HPV16 E7_49-57_ (RAHYNIVTF; R9F). For ELISPOT assays, a control irrelevant peptide was used (WT-_126–134_). All formulations contained a universal T helper peptide PADRE (AKXVAAWTLKAA).

### DPX preparation and administration

Peptides were formulated in DPX with a proprietary immunostimulant as previously described^7^. Peptides and immunostimulant were first solubilized in buffer and mixed 10:1 (w:w) with DOPC/cholesterol mixture (Lipiod GMBH, Germany) to form liposomes. The mixture was then lyophilized to a dry cake which was reconstituted with Montanide ISA51 VG (SEPPIC, France) immediately prior to injection. Mice were injected subcutaneously on the right flank with 50 µl of DPX-R9F containing 10 µg of R9F fused with PADRE + 20 µg of immunostimulant. For experiments with multiple injections, they were given on the same flank, but avoiding previous injection sites. In tumour challenge studies DPX was administered on study days 15 and 29. In immune monitoring studies, DPX was administered on study day 21.

### Cyclophosphamide treatment

CPA was reconstituted in PBS and provided in drinking water at a concentration of 0.133 mg/mL. Based on 3 mL of water/mouse/day, the CPA dose was calculated at 20 mg/kg/day. In tumour challenge studies CPA was provided for one week starting on study day 7 and then again starting on study day 22. In immune monitoring studies, CPA was provided for one week starting on study day 14.

### Antibody treatment

For challenge studies, monoclonal antibodies were administered on study days 15, 18 and 21, and again on study day 29, 32 and 35. For immune monitoring studies, antibodies were administered on study days 21, 24, and 27. Indicated antibodies: Anti-PS (mch1N11, 100 µg/dose), or isotype (C44, 100 µg/dose) and anti-PD-1 (RMP1-14, 200 µg / dose) were given by intraperitoneal injection.

### IFN-y ELISPOT

IFN-γ ELISPOT was performed as previously described^7^. Single cell suspensions were prepared from splenocytes in complete RPMI media (RPMI 1640 (Gibco) + 10% FBS (Hyclone), 2% penicillin/streptomycin (Gibco), 2 mM L-Glutamine (Gibco), 50 mM mercaptoethanol (Sigma-Aldrich), and 5 mM HEPES buffer (Gibco)) and adjusted to 5 × 10^6^ cells/mL. Peptide and splenocytes cells were then added to the IFN-γ ELISPOT plates (BD Bioscience) and incubated overnight at 37 °C in 5% CO_2_ and developed the following day using AEC kit (Sigma Aldrich). Spot forming units were enumerated using the ELISPOT Reader (C.T.L Ltd, Shaker Heights, OH, USA).

### RT-qPCR

Total RNA from tumour tissue was isolated using RNeasy Mini Kit (QIAGEN); 4 µg aliquots were treated with DNase I (Invitrogen) and reverse transcribed using Superscript III reverse transcriptase kit (Invitrogen) and oligo d(T) primer (Invitrogen). Quantitative analysis of transcripts was performed on Rotor-Gene Q real time PCR machine using a QuantiFast SYBR Green PCR kit (QIAGEN). Data were quantified based on the standard curve method and normalized to GAPDH mRNA expression.

### Statistical analysis

Statistical Analysis of data was conducted with GraphPad Prism 6 (La Jolla, CA, USA) software. Data were analysed by appropriate tests as indicated in the figure legends. Significant levels were denoted as: *p < 0.05, ** p < 0.01, *** p < 0.001.

**Figure 1:**
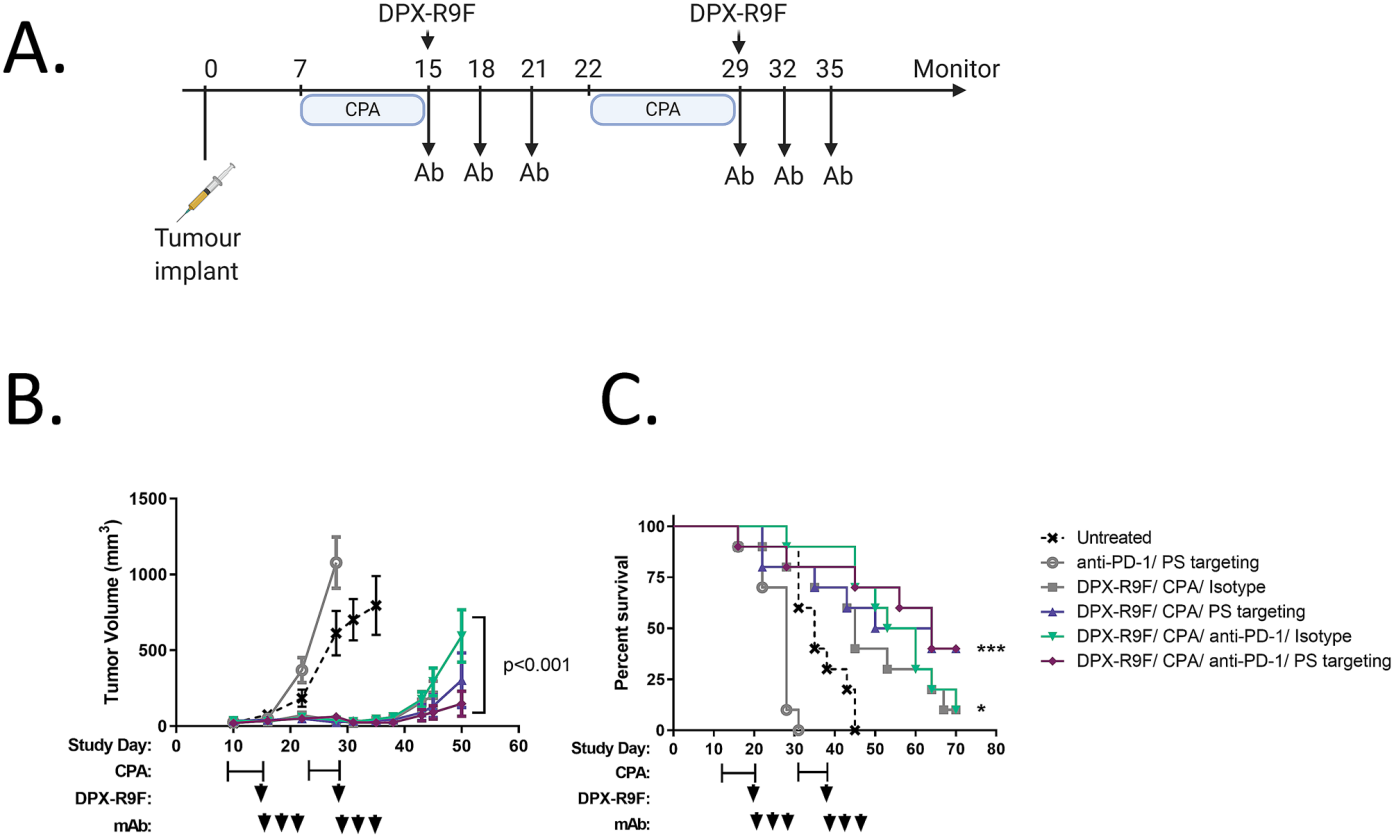
PS Targeting can enhance therapeutic benefit of tumours responsive to immune therapy. (**A**) Study schedule for tumour kinetics and survival: On study day 0, C57BL/6 mice (n = 10) were implanted with HPV16 E7-expressing C3 tumour cells (SC). Groups of mice received treatment with DPX-R9F (containing the peptide epitope HPV16 E7_49-57_) and cyclophosphamide (CPA; 20 mg/kg/day PO), monoclonal antibodies (Ab) PS-targeting (mch1N11; 100 µg/dose IP), C44 and/ or anti-PD-1 (clone RMP1-14; 200 µg/dose IP) as indicated. (**B**) tumour kinetics (**C**) survival. Significance calculated by linear regression for tumour measurements against untreated, and Mantel-Cox compared to untreated for survival studies: *p < 0.05 (DPX-R9F/CPA/anti-PD-1 & DPX-R9F/CPA/isotype), ***p < 0.001 (DPX-R9F/CPA/anti-PS & DPX-R9F/CPA/anti-PS/anti-PD-1).

In tumour challenge studies mice with adverse events such as tumour ulcerations and injection site reactions which resulted in humane endpoint were excluded from the analyses presented in Fig. 1 through 3.

## Results

### Anti-PS combination with DPX-R9F enhances survival and prolongs tumour control over untreated controls

To assess the efficacy of anti-PS targeting in combination of DPX-formulations, we utilized an established model of HPV16 E7-expressing cells injected subcutaneously into C57BL/6 mice. Starting seven days prior to DPX-R9F treatment mice were treated with 7 days of orally administered cyclophosphamide (CPA; 20 mg/kg/day) as previously described^6^. On days 15 and 29, mice were treated with DPX formulated with R9F subcutaneously followed by antibody treatments delivered intraperitoneally on days 15, 18, 21, 29, 32, 35 with anti-PS (mch1N11) or isotype (C44), anti-PD-1 (RMP1-14), or quadruple therapy (DPX-R9F/CPA/anti-PS/anti-PD-1) (Fig. 1A). All groups receiving DPX-R9F and CPA treatment experienced enhanced tumour control compared to both untreated groups and anti-PD-1/anti-PS combination alone (Fig. 1B,C). Interestingly, combination therapy of both anti-PD-1 and anti-PS without DPX-R9F/CPA resulted in increased tumour growth similar to untreated groups, highlighting the benefit of DPX-R9F/CPA treatment in generating immune responses. Previous studies have identified a synergistic effect of combining anti-PS and anti-PD-1 antibodies, however this has not been tested in combination with DPX-R9F treatment in this model^19^. Combination of anti-PD-1 and anti-PS without DPX-R9F did not result in a significant improvement in survival compared to the untreated control group and resulted in a worse outcome possibly due to underlying toxicity issues in this model, and tumour ulcerations which required termination . Within the DPX-R9F-treated groups, the addition of anti-PD-1 prolonged survival and delayed tumour growth over untreated mice but was not significantly different from the isotype control. In contrast, the addition of anti-PS targeting antibodies either alone or in combination with anti-PD-1 resulted in the greatest overall survival (40% survival compared to 10% survival of both DPX-R9F/CPA/isotype or DPX-R9F/CPA/anti-PD-1/isotype), and moderately reduced tumour burden compared to other groups (147.6mm^3^ for DPX/CPA/anti-PS/anti-PD-1 compared to 303.4mm^3^ compared to the DPX-R9F/CPA/anti-PS, and 569.4 mm^3^ for DPX-R9F/CPA/anti-PD-1 at day study day 50). Although much of the differences observed were not statistically significant over isotype controls, or DPX-R9F/CPA alone, due to the reduction in tumour burden compared to formulations lacking DPX-R9F, we sought to characterize the different immune responses generated by each of the combinations and the associated anti-tumour responses.

### Anti-PS did not enhance systemic antigen-specific responses in DPX-R9F treated mice

**Figure. 2:**
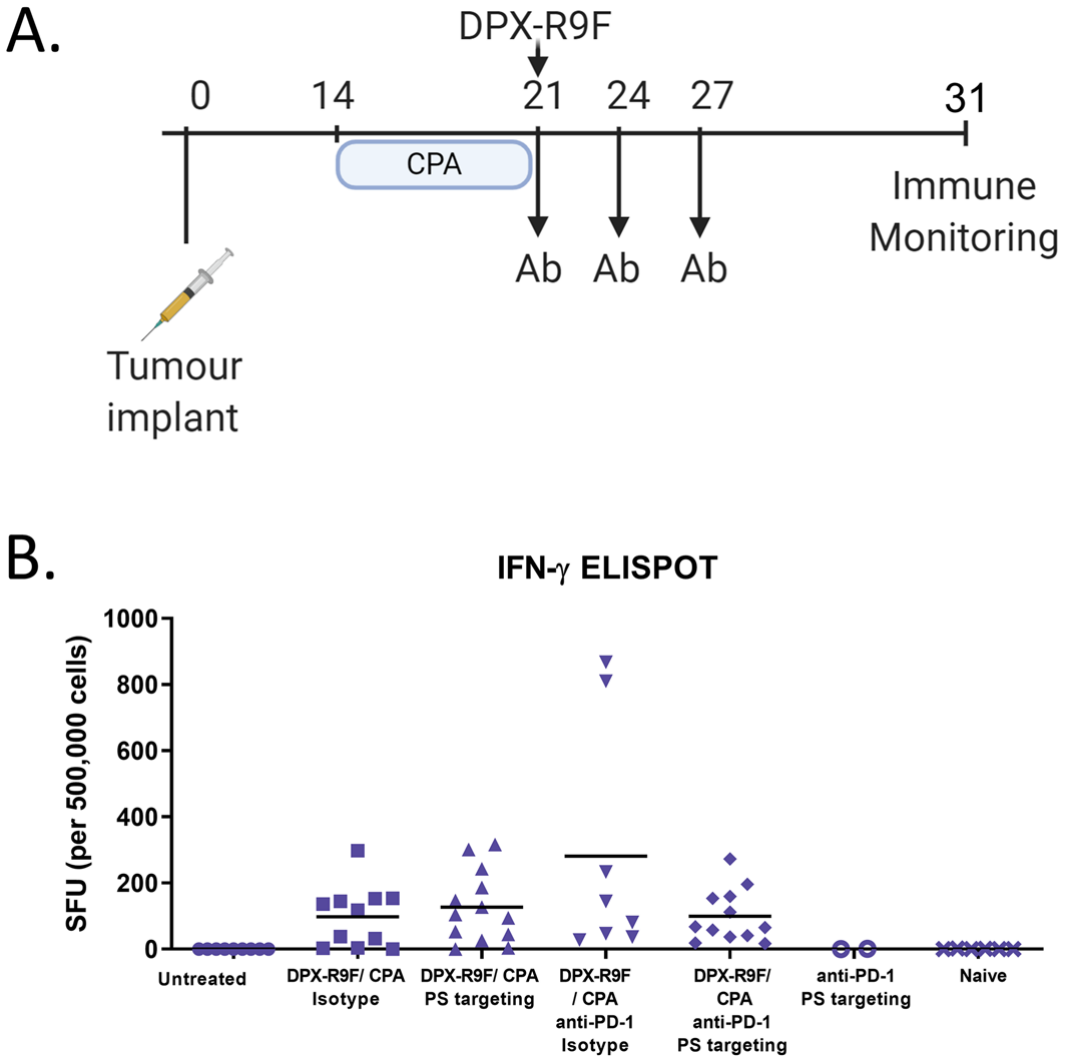
IFN-γ ELISPOT responses within treatment groups. (**A**) IFN-γ ELISPOT was performed using splenocytes (500,000 cells/ well) stimulated with R9F peptide as described in the materials and methods. Groups of mice received treatment with DPX-R9F (containing the peptide epitope HPV16 E7_49-57_) and cyclophosphamide (CPA; 20 mg/kg/day PO), monoclonal antibodies (Ab) PS-targeting (mch1N11; 100 µg/dose IP), C44 and/ or anti-PD-1 (clone RMP1-14; 200 µg/dose IP) as indicated. (**B**) Results shown have background removed. No significant difference was detected between DPX-R9F-treated groups using one-way ANOVA followed by a Tukey post test.

The efficacy of many cancer therapeutics is based around the specific targeting of tumour antigens^23,24^. Due to the unique ability of DPX to harness antigen-presenting cells and direct antigen specific T cell responses^5^, we sought to examine this phenomenon in combination with anti-PS antibodies as a possible mechanism of enhanced tumour control and survival. To test the ability of anti-PS treatment to affect antigen-specific responses, spleens were harvested from tumour-bearing treated mice, and subjected to an IFN-γ ELISPOT assay on study day 31 (Fig. 2A). The control untreated and non-tumour bearing naïve mice failed to elicit antigen specific responses in the IFN-γ ELISPOT Fig. 2B. In contrast, antigen specific responses were observed in all treatment groups which included DPX-R9F, highlighting the ability of DPX to harness antigen-specific responses. No increases in antigen-specific responses were observed in the anti-PD-1/anti-PS group, however, a limited sample size precluded proper comparison. No significant difference was observed between any of the DPX-R9F combinations over their respective isotype control treatment groups.

### DPX-R9F/CPA/anti-PS upregulates expression of genes associated with cytotoxicity and antigen presentation

Not all tumour-specific responses can be directly observed within circulating PBMCs and detected by IFN-γ ELISPOT^25^. Therefore, we directed our analysis to intra-tumour characterization of the responses. Previous studies examining the mechanism of action of anti-PS treatment demonstrated enhanced tumour infiltration of immune cells^21,26^. In line with the mechanism of action of anti-PS, we continued to assess the tumour micro environment using RT-qPCR. In this analysis HPV16 E7-expressing cells were implanted subcutaneously in the left flanks of C57BL/6 mice, CPA was administered orally 7 days prior to DPX-R9F treatment on day 21. Anti-PS or isotype, anti-PD-1, or anti-PS/anti-PD-1 combination was administered on day 21, 24 and day 27 (Fig. 2A). All mice were terminated on day 31 and tumours were extracted and analyzed by RT-qPCR.

**Figure 3:**
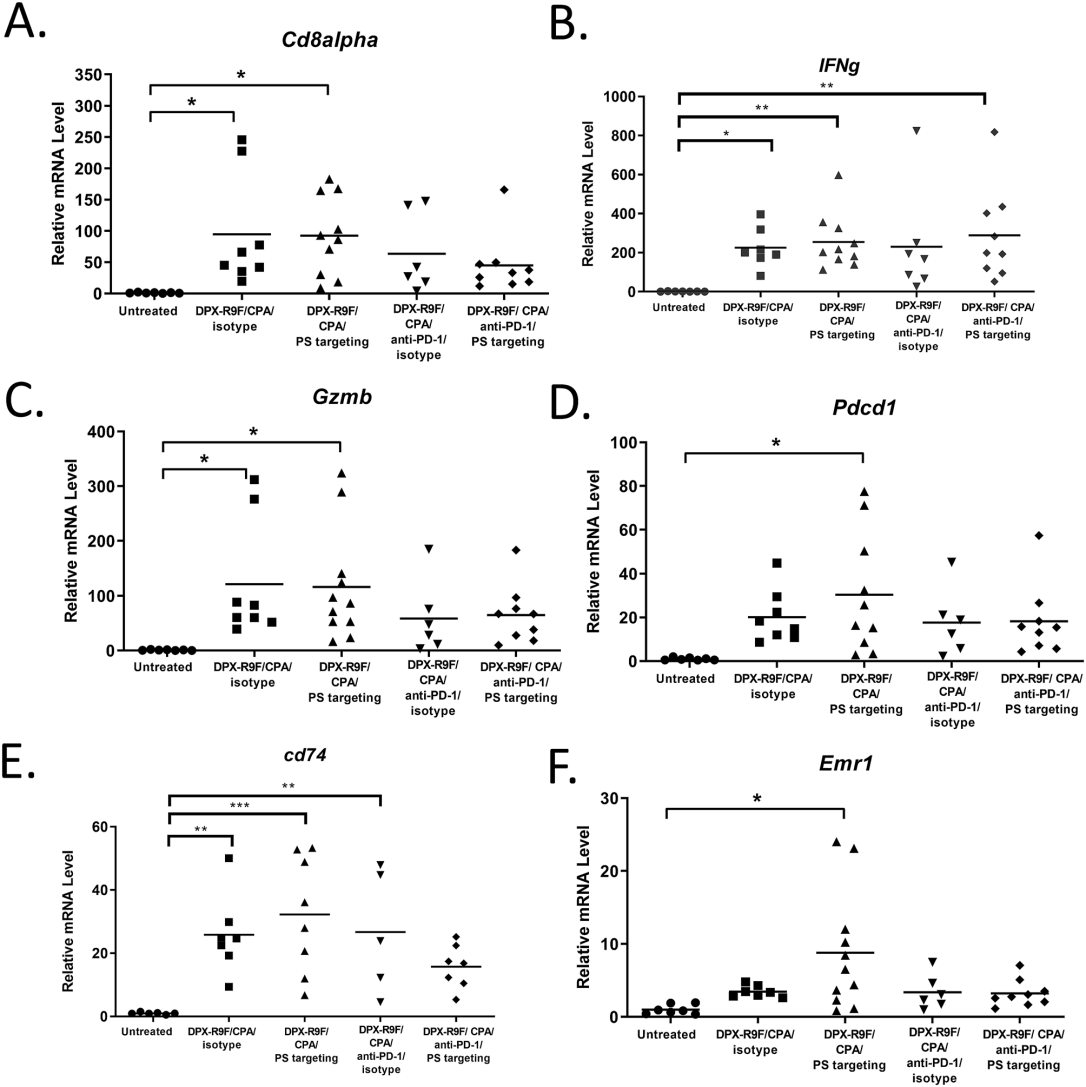
Increases in cytotoxic CD8α^+^ T cells and antigen presenting cells within the tumour microenvironment confirmed by RT-qPCR. C57BL/6 mice (n = 5–11) were treated as in Fig. 2, data pooled from two separate experiments. Tumours were analyzed by RT-qPCR for expression of (**A**) *Cd8alpha* (CD8a), (**B**) *IFNγ*. (**C**) *Gzmb* (Granzyme B), (**D**) *Pdcd1* (PD-1), (**E**) *Cd74* (**F**) *Emr1* (F4/80) Statistics by one-way ANOVA followed by Tukey post test: *p < 0.05 *p < 0.01, ***p < 0.001.

To evaluate changes induced by treatment in the tumour microenvironment we analyzed expression of genes associated with immune cell infiltration and activation by RT-qPCR (*cd8α*, *gzmb*, *tnfα*, *pdcd1, cd74*, *cd80, F4/80*, *ifng* and *cd68*). Analysis of tumours from DPX-R9F/CPA/anti-PS treatment resulted in elevated expression of *cd8α* compared to untreated controls (Fig. 3A). Similar increases were detected in the DPX-R9F/CPA/isotype treatment group, highlighting the effectiveness of DPX-R9F treatment in enhancing tumour infiltrating lymphocytes. Although modest increases in *cd8a* were detected in both anti-PD-1 treatment groups (DPX-R9F/CPA/anti-PD-1/isotype or anti-PS), these differences were not significant from the untreated control. Consistent with the elevated levels of *cd8α* mRNA, increased levels of cytotoxic markers IFN-γ (*ifng*) and the cytolytic granule enzyme, granzyme B (*gzmb*) were found in both DPX-R9F/CPA/isotype or anti-PS groups (Fig. 3A,C). In the tumours treated with DPX-R9F/CPA/anti-PD-1 the levels of *gzmB* and *ifng* transcripts were slightly elevated but not statistically different from the levels in the untreated control group (Fig. 3B,C). Elevated levels of PD-1 (*pdcd1*) transcripts were observed in the DPX-R9F/CPA anti-PS group, but not in treatment groups containing anti-PD-1 (Fig. 3D). Quadruple therapy resulted in suppressed immune cell gene signatures within the tumour like untreated levels, indicating that anti-tumour responses may be driven through different mechanisms than strictly immune cell infiltration.

We next assessed expression of *cd74*, an HLA-DR gamma protein that plays a critical role in MHC class II-mediated antigen presentation in macrophages and dendritic cells^27^. All treatment groups other than the quadruple DPX-R9F/CPA/anti-PD-1/anti-PS group had significantly elevated levels of *cd74* compared to untreated controls (Fig. 3E). Furthermore, RT-qPCR revealed a marked increase in the number of F4/80 (*emr1*) transcripts in the DPX-R9F/CPA/anti-PS treated group compared to untreated and isotype controls (Fig. 3F). Taken together, these results highlight the capability of anti-PS treatment to enhance tumour infiltration of both cytolytic, and antigen-presenting cell types. The addition of the anti-PD-1 to the treatment regime appears to slightly improve tumour control, but by a different mechanism than directly enhanced immune cell infiltration within the tumour.

## Discussion

A major hurdle to over come when implementing tumour targeted immunotherapy is the intrinsic immunosuppressive nature of many tumour microenvironments. One such mechanism is the presence of PS on the surface of malignant cells, microvesicles and apoptotic bodies that are naturally immunosuppressive in nature^20^. Recently, a blockade of PS through binding of surface exposed PS has proven to be an effective way at generating anti-tumour immunity by targeting the tumour microenvironment itself^26,28,29^. In the present study we demonstrate enhanced tumour control and prolonged survival of tumour bearing mice when DPX-R9F immunotherapy is combined with anti-PS or anti-PS and anti-PD-1 combination antibody treatment over untreated controls (Fig. 1B,C). Upon investigation into the underlying immune responses of different treatment groups, we identified that treatment of DPX-R9F with anti-PS did not enhance peripheral antigen-specific responses as determined by IFN-γ ELISPOT (Fig. 2B), but resulted in elevated numbers of CTL, dendritic cell and macrophage gene signatures within the tumour. Upon addition of anti-PD-1 antibodies combined with DPX-R9F/anti-PS, there was a significant decrease in the expression of immune cell markers, suggesting that anti-PD-1 has the ability to systemically alter the status of immune cells^30^, whereas anti-PS can directly influence the TME^20^. Overall, these results highlight differing mechanisms by which various combination therapies with DPX can modulate the intra-tumoural, and systemic immune responses within our model.

Treatment of cancer in vivo and in vitro with anti-PS treatment have highlighted the role of antigen-presenting cells to specifically target cancerous cells^20,29^. These findings were characterized as increased infiltration upon treatment to initiate immune responses and tumour cell engulfment^31^. Here, we recapitulate these findings when combining DPX-R9F with anti-PS by RT-qPCR analysis of tumour tissue, where gene signatures associated with APCs are enhanced with treatment. Not directly studied here was the role of NK cells in tumour cell lysis which can also be enhanced through tumour specific antibodies such as anti-PS^32,33^. It is likely that NK cell-mediated ADCC plays an import part in controlling tumour control in the anti-PS treated groups, and warrants further investigation within this model.

The addition of anti-PD-1 to DPX-R9F/CPA/anti-PS therapy resulted in a decreased expression of both antigen-presenting cell markers and cytolytic cell markers within the tumour compared to DPX-R9F/CPA/anti-PS treatment. Interestingly, the drop in expression of classical anti-tumour genes (*ifng* and *gzmb*) did not result in any worse tumour burden or decreased survival (Fig. 1). During the immune analysis, only one timepoint was analyzed (SD31), and may not have been the optimum timepoint to observe the changes between all groups. It is possible that combination of all three agents targeting different aspects of the anti-tumour response, generation of tumour-specific T cells, enhancing functionality of T cells, and direct tumour targeting, may have resulted in delayed or accelerated lymphocyte infiltration which was not detected in this assay. Further longitudinal analysis may be required to fully elucidate the mechanism governing the synergistic effects of combination treatment.

DPX is a novel T cell activating platform which allows for active uptake of tumour-specific antigens by antigen presenting cells^4^. Combination of DPX-based immunotherapy with other immunomodulatory agents such as CPA and anti-PD-1 has yielded promising therapeutic capabilities^6,7^. Although survival was not significantly different from single antibody treatment, dual antibody treatment (anti-PS and anti-PD-1) in combination with DPX-R9F/CPA exhibited some level of tumour control compared to untreated, and also resulted in differing immune responses which seemingly allowed for tumour control similar to that of the other DPX-R9F/CPA-containing groups (Fig. 1B,C). Although, we were unable to determine a definitive mechanism for this phenomenon, it can likely be attributed to a cumulation of multiple converging anti-tumour effects. These results are akin to our previous studies evaluating anti-PD-1 in combination with DPX-R9F formulations, where we observed limited tumour infiltration of immune cells but significant expansion of CTLs in the spleen^7^. Moreover, antigen-specific clones were found to have expanded TCR-β repertoires toward R9F, and correlated to tumour control^7^. Paradoxically, it remains possible that addition of anti-PD-1 to the DPX-R9F/CPA/anti-PS hindered the classical anti-tumour responses. Compared to the DPX-R9F/CPA/anti-PS treatment, the addition of PD-1 lowered overall infiltration of immunes cells determined by RT-qPCR, and had a slight reduction in ELISPOT responses. Despite these differences, the quadruple therapy did not decrease survival or tumour burden significantly from the triple therapy with anti-PS.

We have previously demonstrated the benefit of anti-PD-1/CPA/DPX-R9F in this tumour model^7,34^. However, we are unable to observe an additive effect when including anti-PS to the therapy. In future experiments, it would be prudent to evaluate different timelines for administration of anti-PD-1 or anti-PS. Recently it has been demonstrated in both preclinical and clinical studies that timing of PD-1 may impact the generation of dysfunction T cells should sufficient priming of T cells following a vaccination of cancer peptides not take place^35^. In line with these recent findings, we have administered DPX and anti-PD-1 simultaneously to minimize this effect. However, combination of PD-1 with various cancer-targeting antibodies may require a further delay in anti-PD-1 administration to see maximum benefit, and greatest tumour control^36^. Incorporating these findings in the management of a combination antibody approach warrant further investigation to tease out how checkpoint inhibitors play a role in this novel combination therapy.

In conclusion, the addition of anti-PS to the DPX-R9F/CPA therapy in the HPV16 E7-expressing tumour model resulted in enhanced anti-tumour responses, resulting in modest levels of tumour control compared to untreated and isotype control groups. The addition of anti-PD-1 to the treatment did offer enhanced survival compared to isotype control, and was not significantly different compared to DPX-R9F/CPA/anti-PS. Whether or not APCs drive the infiltration of other leukocytes, remains an active area of research, and will require in vivo mouse models to functionally link APC chemotactic factors to lymphocyte recruitment. The anti-PS blockade is an efficient method to target the tumour microenvironment, and the results presented here warrant further investigation of tumour targeting agents with novel T cell activating therapies.

### Ethical approval

All experimental protocols were approved by the university committee on laboratory animals at Dalhousie University under the guidelines of the Canadian Council on Animal Care ethical standards. All applicable international, national, and institutional guidelines for the care and use of animals were followed. This article does not contain any studies with human participants performed by any of the authors.
